# Effect of span length and connector size on fracture resistance of monolithic zirconia FPDs: A finite element analysis

**DOI:** 10.6026/973206300212523

**Published:** 2025-08-31

**Authors:** Mahitosh Lakhe, Akshay Bhargava, Rajiv Kumar Gupta, Bharti Dua, Cherry Anmol, Manisha Yerson

**Affiliations:** 1 Department of Prosthodontics and Crown & Bridge, Santosh Dental College and Hospital, Santosh Deemed To Be University, Ghaziabad, Uttar Pradesh, India

**Keywords:** Monolithic zirconia, fixed partial denture (FPD), edentulous span length, connector size, finite element analysis (FEA), fracture resistance, stress distribution

## Abstract

The effect of span length and connector size on the fracture resistance of monolithic zirconia fixed partial dentures (FPDs). Twelve mandibular FPD models with 3-, 4-, and 5-unit spans (9 mm, 16 mm, and 23 mm) were digitally created, each incorporating connector sizes of 12 mm^2^, 15 mm^2^, 18 mm^2^ and 21 mm^2^. Finite element analysis using ANSYS 2024 simulated physiological (324 N) and maximal (1270 N) occlusal forces. Longer spans with smaller connectors generated stresses exceeding zirconia's tensile strength, while larger connectors reduced stress values significantly. Data shows that optimizing connector size is essential to improve biomechanical performance and longevity of zirconia FPDs, especially in long-span restorations.

## Background:

The widespread adoption of zirconia in fixed dental prostheses (FPDs) is driven by its superior mechanical properties, biocompatibility, and esthetic advantages over traditional metal-ceramics [1]. Monolithic zirconia, in particular, eliminates the need for veneering ceramics, thereby reducing the risk of chipping and enhancing longevity [2]. However, despite its high flexural strength and fracture toughness, zirconia restorations remain susceptible to structural failure under functional load, especially in long-span prostheses [3, 4]. One of the most critical determinants of fracture resistance in zirconia FPDs is the design of the connector-the area that joins pontics to retainers. Inadequate connector dimensions can result in stress concentrations that surpass zirconia's tensile limits, leading to catastrophic failure [5]. Both in vitro experiments and clinical data indicate that increasing connector cross-sectional area significantly enhances the fracture load capacity of FPDs [6]. Moreover, span length directly influences stress distribution, with longer spans amplifying flexural stresses across connectors [7]. Finite element analysis (FEA) has become an indispensable tool in evaluating the biomechanical behavior of dental materials and prosthesis designs. It allows for precise simulation of functional forces and material responses without the variability of clinical trials [8]. Previous FEA studies have demonstrated that optimizing connector geometry and span configuration can significantly improve the stress distribution and mechanical performance of zirconia FPDs [9]. Ogino and Nomoto (2016) investigated how variations in connector design influence the fracture resistance of zirconia-based fixed partial dentures in the upper anterior region, emphasizing the importance of optimizing connector geometry to balance strength with esthetic requirements [10]. Therefore, it is of interest to evaluate, using finite element methods, how variations in span length and connector size influence the stress patterns and potential failure zones in monolithic zirconia FPDs.

## Methodology:

The study aimed to evaluate the effect of connector size on the mechanical performance of monolithic zirconia fixed partial dentures (FDPs) using finite element analysis (FEA). To simulate realistic clinical conditions, mandibular arch models were fabricated from Type IV dental stone to represent three edentulous span lengths: short (9 mm), medium (16 mm), and long (23 mm). These models supported 3-unit, 4-unit, and 5-unit FDPs, replacing one, two, and three missing posterior teeth, respectively. The models were scanned using a Shining 3D extraoral scanner, which produced high-resolution STL files capturing the morphology of the edentulous spans and abutment teeth. Digital bridge frameworks were designed in EXOCAD software, with four connector cross-sectional areas-12 mm^2^, 15 mm^2^, 18 mm^2^, and 21 mm^2^-integrated into each span length. All connectors featured a circular profile with a 0.9 mm gingival embrasure radius to reduce stress concentration. The designs were exported to Autodesk Fusion 360 for preliminary checks and then refined in Rhinoceros 3D, where meshing was performed using tetrahedral elements to capture anatomical complexity. Mesh convergence tests ensured that stress variations remained within 5% across refinements. Final models were imported into ANSYS Workbench 2024 for static structural analysis. Material properties were assigned based on literature values: zirconia was modeled with an elastic modulus of 210 GPa, Poisson's ratio of 0.31, and density of 6.05 g/CM^3^; porcelain with 70 GPa and 0.19; and dentin with 18.6 GPa and 0.31. All materials were treated as homogeneous, isotropic, and linearly elastic. The supporting teeth were fixed at the base to mimic osseointegration, and vertical forces ranging from 324 N to 1270 N were applied to the central fossa of each pontic over a 2 mm^2^ area, representing physiologic masticatory loading. Perfect bonding was assumed between zirconia, porcelain, and tooth structures to ensure smooth force transfer. Von Mises stress distributions were analyzed, particularly at the gingival connector areas, and both maximum and average stress values were recorded. Statistical analysis using the Kruskal-Wallis and Mann-Whitney U tests was conducted to evaluate the influence of connector size and span length on stress distribution, with significance set at p < 0.05.

## Results:

Finite element analysis (FEA) was performed using ANSYS 2024 to investigate the von Mises stress distributions in 12 mandibular 3,4,5-unit fixed partial denture (FDP) comprising three edentulous span lengths (9 mm, 16 mm, 23 mm) and four connector cross-sectional areas (12 mm^2^, 15 mm^2^, 18 mm^2^, 21 mm^2^). The FDP models, constructed with 3 vol% yttria-stabilized tetragonal zirconia polycrystal (Y-TZP), were meshed with tetrahedral elements, yielding node counts of 135,000 to 141,000 and element counts of 72,000 to 75,200, as detailed in Table 1 (see PDF). Vertical occlusal loads of 324 N and 1270 N were applied at the central fossa of the pontic to simulate normal mastication and maximum clenching forces, respectively. This section presents the maximum von Mises stress results for each FDP design under the two loading conditions, organized by span length. The findings elucidate the biomechanical performance of the FDPs, addressing the study's objective to optimize connector dimensions for enhanced fracture resistance in monolithic Y-TZP restorations. To facilitate integration into the thesis, the results are described textually, with recommendations provided for generating graphical representations. The FDP designs with a 9 mm edentulous span exhibited the lowest maximum von Mises stresses, attributable to the reduced bending moments associated with shorter spans. The stress values for the four connector areas under 324 N and 1270 N loads are given in Table 1 (see PDF). The stresses, ranging from 34 MPa to 235 MPa, were substantially below the tensile strength of Y-TZP (900 MPa), indicating no risk of material failure across all 9 mm span designs, even under maximum clenching forces. The 16 mm span FDP designs, representing an intermediate span length, demonstrated increased stresses due to greater bending moments. The maximum von Mises stresses for the four connector areas under 324 N and 1270 N loads are given in Table 2 (see PDF). The stresses ranged from 107 MPa to 736 MPa. Notably, the 12 mm^2^ connector under 1270 N produced a stress of 736 MPa, approaching the Y-TZP tensile strength of 900 MPa, suggesting a potential risk of fracture under extreme loading conditions. The 18 mm^2^ and 21 mm^2^ connectors maintained stresses below 500 MPa under 1270 N, indicating enhanced structural reliability. The 21 mm^2^ connector reduced stress by approximately 43% compared to the 12 mm^2^ connector. The stress values for the four connector areas under 324 N and 1270 N loads are given in Table 3 (see PDF).

## Discussion:

The results of this finite element study underscore the critical influence of both span length and connector size on the biomechanical performance of monolithic zirconia fixed partial dentures (FPDs). The data demonstrated that increasing the connector cross-sectional area from 12 mm^2^ to 21 mm^2^ significantly reduced Von Mises stress values, thereby improving fracture resistance across all span configurations. This finding aligns with previous in vitro studies, where larger connectors consistently exhibited higher fracture load thresholds in zirconia prostheses [11, 12]. Longer-span prostheses (particularly 5-unit FPDs with a 23 mm pontic span) displayed stress concentrations that exceeded the tensile strength of zirconia when paired with smaller connectors (12 mm^2^), confirming that span length is a compounding risk factor for structural failure. Subsomboon and Urapepon (2023) similarly reported that longer spans and narrow connectors reduce load-bearing capacity, even in high-strength materials like zirconia [11]. Azmin *et al.* (2023) further demonstrated that connector geometry, especially height and radius of curvature, plays a critical role in load transfer, where abrupt transitions can exacerbate stress concentrations and increase fracture risk [12]. Another factor influencing fracture behavior is the intrinsic material properties of zirconia. While monolithic zirconia offers higher strength compared to bilayered ceramics, it is still susceptible to low-temperature degradation and fatigue over time, particularly under cyclic occlusal loading [13]. The durability of zirconia frameworks is therefore dependent not only on connector dimensions but also on careful design to avoid stress-risers, particularly at gingival embrasures, as highlighted by Sundh *et al.* (2005) [13]. Interestingly, while increasing connector size improves fracture resistance, it may also impose esthetic or anatomical limitations, especially in posterior regions with limited vertical space. Ardakani *et al.* (2019) emphasized that framework design must balance mechanical integrity with soft tissue and esthetic considerations, reinforcing the clinical relevance of careful connector planning [14]. From a clinical standpoint, monolithic zirconia FPDs has shown favorable medium- to long-term outcomes when designed with robust connectors. Habibi *et al.* (2020) reported high survival rates over three years, noting that structural failures were predominantly linked to undersized connectors or suboptimal design [15]. Likewise, Prott *et al.* (2021) and Spitznagel *et al.* (2022) documented improved fatigue behavior and failure loads in prostheses with increased thickness and optimized connector dimensions [16, 17]. The implications of these findings extend to implant-supported prostheses as well. As shown by Spitznagel *et al.* (2022), implant-borne zirconia FPDs are even more susceptible to stress-induced failures due to the absence of periodontal ligament damping. This makes the role of connector design even more significant in such restorations [17]. Systematic reviews, such as Raigrodski *et al.* (2012), emphasize that a significant proportion of mechanical failures in zirconia-based FPDs are connector-related, highlighting the necessity of evidence-based design principles [18]. Hjerppe *et al.* (2025) also support the use of monolithic zirconia in posterior 3-unit FPDs, with one-year interim data showing promising survival rates-provided that connector size and framework configuration are appropriately planned [19]. Zhang and Lawn (2018) demonstrated that the fracture resistance of zirconia restorations is significantly influenced by structural design factors, including connector dimensions and span length. Their findings suggest that optimizing connector size is critical to minimizing tensile stresses and enhancing the longevity of monolithic zirconia FPDs [20]. Thus, this study validates prior research by showing that both connector size and span length significantly impact stress distribution in zirconia FPDs. Connector dimensions of at least 18-21 mm^2^ are recommended, particularly for longer-span restorations, to maintain structural reliability. These findings can guide clinicians and dental technicians in designing more durable, fatigue-resistant zirconia prostheses.

## Conclusion:

This study demonstrated that edentulous span length and connector size significantly affect stress distribution and fracture resistance in monolithic Y-TZP fixed partial dentures. Longer spans and smaller connectors increased stress, especially under high occlusal loads, while circular connectors' ≥18 mm^2^ enhanced structural integrity. These findings offer evidence-based guidance for optimizing FDP design to improve clinical performance.
